# Metagenome phylogenetic profiling of microbial community evolution in a tetrachloroethene-contaminated aquifer responding to enhanced reductive dechlorination protocols

**DOI:** 10.1186/s40793-016-0209-z

**Published:** 2016-12-01

**Authors:** Rebecca A. Reiss, Peter Guerra, Oleg Makhnin

**Affiliations:** 1 New Mexico Institute of Mining and Technology, 801 Leroy Place, Socorro, NM 87801 USA; 2 AMEC Foster Wheeler Environment & Infrastructure, Inc, 8519 Jefferson NE, Albuquerque, NM 87113 USA

**Keywords:** Enhanced reductive dechlorination, Metagenomics

## Abstract

Chlorinated solvent contamination of potable water supplies is a serious problem worldwide. Biostimulation protocols can successfully remediate chlorinated solvent contamination through enhanced reductive dechlorination pathways, however the process is poorly understood and sometimes stalls creating a more serious problem. Whole metagenome techniques have the potential to reveal details of microbial community changes induced by biostimulation. Here we compare the metagenome of a tetrachloroethene contaminated Environmental Protection Agency Superfund Site before and after the application of biostimulation protocols. Environmental DNA was extracted from uncultured microbes that were harvested by on-site filtration of groundwater one month prior to and five months after the injection of emulsified vegetable oil, nutrients, and hydrogen gas bioamendments. Pair-end libraries were prepared for high-throughput DNA sequencing and 90 basepairs from both ends of randomly fragmented 400 basepair DNA fragments were sequenced. Over 31 millions reads were annotated with Metagenome Rapid Annotation using Subsystem Technology representing 32 prokaryotic phyla, 869 genera, and 3,181 species. A 3.6 log_2_ fold increase in biomass as measured by DNA yield per mL water was measured, but there was a 9% decrease in the number of genera detected post-remediation. We apply Bayesian statistical methods to assign false discovery rates to fold-change abundance data and use Zipf’s power law to filter genera with low read counts. Plotting the log-rank against the log-fold-change facilitates the visualization of the changes in the community in response to the enhanced reductive dechlorination protocol. Members of the *Archaea* domain increased 4.7 log_2_ fold, dominated by methanogens. Prior to remediation, classes *Alphaproteobacteria* and *Betaproteobacteria* dominated the community but exhibit significant decreases five months after biostimulation. *Geobacter* and *Sulfurospirillum* replace “*Sideroxydans*” and *Burkholderia* as the most abundant genera. As a result of biostimulation, *Deltaproteobacteria* and *Epsilonproteobacteria* capable of dehalogenation, iron and sulfate reduction, and sulfur oxidation increase. Matches to thermophilic, haloalkane respiring archaea is evidence for additional species involved in biodegradation of chlorinated solvents. Additionally, potentially pathogenic bacteria increase, indicating that there may be unintended consequences of bioremediation.

## Introduction

Contamination of groundwater with chlorinated solvents is a major threat to potable water supplies, but the challenge of remediating contamination can be addressed by exploiting the metabolism of microbial communities. While qPCR and microarrays are effective tools for designing and monitoring remediation strategies [1–3], whole metagenome phylogenetic profiling provides unparalleled opportunities to understand the genetic response of microbial communities to remediation strategies. A proof-of-concept project was undertaken to determine the efficacy of whole metagenome profiling of aquifer-borne microbial consortia in a tetrachloroethene-contaminated EPA superfund site to increase our understanding community response to biostimulation.

Tetrachloroethene (also known as perchloroethylene) is a solvent used for dry cleaning since the 1930s. PCE is now a common contaminant of groundwater that is a likely carcinogen [4]. The daughter products of PCE biodegradation include TCE, cis and trans isomers of DCE, VC, and ethene. VC is the most toxic of these compounds and a major problem is the stalling of biodegradation. Enhanced *in*-*situ* biodegradation involves the addition of electron donor or food source (substrate), electron acceptors, nutrients, and/or selective cultures of beneficial microbes into the subsurface to accelerate the rate of biodegradation. RD is a biodegradation process that is restricted to anaerobic conditions capable of eliminating PCE and its daughter products. The selection of appropriate electron donors is an important site-specific component for ERD. Although our current knowledge of the microbes and the biochemical pathways involved RD is largely limited to species that can be cultured, reductive anaerobic biological in-situ treatment technology augmentation protocols create favorable environmental conditions for RD microbial consortia [5]. This study compares two whole metagenomes from the same well within a PCE-contaminated aquifer one month before and five months after biostimulation with electron donors and nutrients. This is part of a larger study that includes two additional time points (23 and 43 months) for this well, and similar points for an additional well on the NRAP site.

### Site information

In 1989, PCE and TCE were detected in municipal water supply wells operated by the City of Española, New Mexico. The source of the contamination was a 58-acre (0.23 km^2^), 260-foot (79.2 m)-deep plume of PCE from a now closed dry cleaner and laundromat located on North Railroad Avenue. Because this aquifer is the sole source drinking water aquifer for Española, the Santa Clara Pueblo, and nearby populations, the NRAP site was designated an EPA Superfund site in 1999 (National Priorities List #NMD986670156). Some minor soil contamination was found near the PCE surface release at NRAP; however, most of the contaminant mass was in the shallow saturated zone and occurred as high-level, adsorbed- and dissolved-phase PCE in the aquifer beneath the release area (source area). Prior to remedy implementation the dissolved-phase plume migrated to within the boundaries of the Santa Clara Pueblo trust lands above maximum allowable contaminant levels.

The selection of a remediation strategy relied on the analysis of groundwater using established regulatory guidelines for geochemical and contaminant chemistry [6], and commercially available molecular biological techniques [7]. As per ITRC protocols water quality parameters such as dissolved oxygen, temperature, pH, oxidation-reduction potential, and specific conductance were monitored in the field. Laboratory analyses included a suite of geochemical parameters shown in Table 10. Data from DGGE of 16S rRNA genes, PLFA analysis, and a qPCR screen for dechlorinating bacteria, was obtained from Microbial Insights, Inc. (Rockford, TN). Genera detected by DGGE included *Dechloromonas*, *Sulfurimonas*, *Thiomicrospira*, *Sulfurovum*, *Gallionella*, and *Zoogloea*. PLFA analysis indicated the presence of *Firmicutes* and anaerobic metal reducing bacteria. The presence of indigenous dechlorinating genera detected by qPCR included *Dehalococcoides*, *Desulfuromonas*, and *Dehalobacter*, indicating that the addition of non-indigenous microbes (known as bioaugmentation) was unnecessary. This data indicated the appropriate remedial action for NRAP was biostimulation, achieved by injecting the bioamendments EVO, a nutrient mix, and the addition of hydrogen gas as an electron donor [7].

**Fig. 1:**
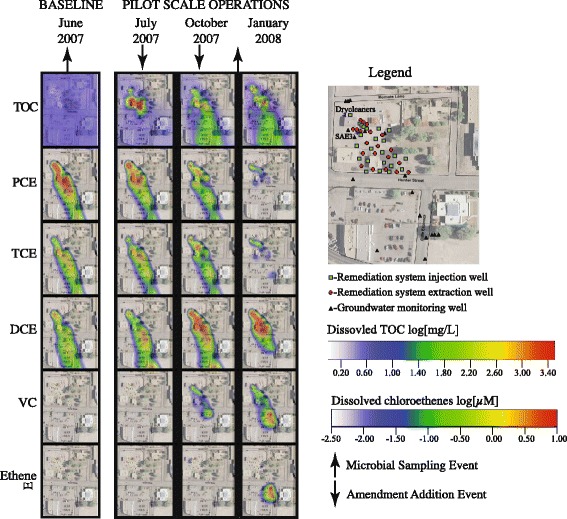
Heat map of TOC and VOCs during pilot scale operations at NRAP. Up- arrows indicate when microbial samples were collected; down arrows indicate when bioamendments were injected into the aquifer

Figure 1 shows the progress of VOC conversions during pilot scale remediation operations at NRAP. Prior to the addition of bioamendments, the majority (~97%) of the chloroethenes were in the form of PCE. TCE (~1.0%) and DCE isomers (~1.5%) accounted for the remaining mass; and VC was not detected in any of the wells sampled. By comparison, in October 2007, a large majority of the contaminant mass was transformed from PCE to cis-DCE. More than three-quarters (~77%) was cis-DCE while 20% remained as PCE. TCE and VC accounted for the remaining portion at 1.5% each. Ethene production was evident by January 2008.

## Metagenome sequencing information

### Metagenome project history

NRAP was sampled four times for nucleic acid analysis starting in 2007; once one month prior to, then at five, 23, and 43 months following the addition of bioamendments. The two time points selected for this preliminary study were during the pilot-scale operations at NRAP, one collected prior to the initiation of bioremediation and one collected five months after the initial injections of EVO, nutrient mix, and H_2_ (Fig. 1). Study information is shown in Table 1.

**Table 1:** Study information

Label	SAE3-0	SAE3-5
MG-RAST ID	EW3Pre1 (4447797.3) EW3Pre2 (4447834.3)	EW3Post1 (4447837.3) EW3Post2 (4447838.3)
SRA ID or ENA ID	NA	NA
Study	North Railroad Avenue Plume EPA Superfund Site	North Railroad Avenue Plume EPA Superfund Site
GOLD ID (sequencing project)	NA	NA
GOLD ID (analysis project)	NA	NA
NCBI BIOPROJECT	NA	NA
Relevance	Bioremediation of contaminated groundwater	Bioremediation of contaminated groundwater

### Sample information

The sampling events analyzed as part of the pilot project occurred on 15 June 2007 (SAE3-0) and on 27 November 2007 (SAE3-5) at the NRAP site in Española, NM (latitude: 35.992053, longitude: −106.079752, and a depth of 2.9 m.) Sample information is provided in Table 2 as per minimal information standards [8]. The exact time of sampling was not recorded, but both samples were extracted from the well in the afternoon, at approximately 14 h local time.

**Table 2:** Sample information

Label	SAE3-0	SAE3-5
GOLD ID (biosample)	NA	NA
Biome	Freshwater	Freshwater
Feature	Superfund site	Superfund site
Material	Ground water	Ground water
Latitude and Longitude	35.992056, −106.07975	35.992056, −106.07975
Vertical distance	−2.9 m	−2.9 m
Geographic location	Española, New Mexico, USA	Española, New Mexico, USA
Collection date and time	13/06/07, 14 h (UTC-6)	27/11/07, 14 h (UTC-7)

## Sample preparation

Suspended solids, including microbes were collected on-site by filtering the water through a Gelman 0.2 micron filter capsule (Cat. #12117, Pall Gelman, Ann Harbor, MI) attached to the discharge of a dedicated, submersible pump for SAE3. The volume of water filtered was monitored and filtration was allowed to proceed until the flow was reduced due to clogging of the filter. Filters were sealed with entrained water and residue and transported to the laboratory on ice. Filter residues, inclusive of captured microbes and particulates, were recovered by shaking the filters overnight on a Berrell wrist-action shaker (Model 75, Philadelphia, PA) followed by back flushing the filter with sterile 10 mM Tris-SO_4_ at a pH 7.8. Samples were concentrated by centrifugation at 25,000 x g for 30 minutes. The resulting pellet was resuspended in Tris-SO_4_ and frozen at −20 °C without cryopreservation for nucleic acid extraction.

### DNA extraction

Microbial eDNA was extracted from the uncultured but concentrated microbial samples SAE3-0 and SAE3-5 using protocols for microbes pursuant to the G-nome® DNA isolation kit (Qbiogene Cat # 2010–200, Carlsbad, CA), consisting of treatment with RNase, lysis of the cells, protease treatment, and precipitation of the protein and lipids with high salt. The eDNA in the resulting supernatant was collected and precipitated by the addition of 1/10 volume of 3 M sodium acetate and 2.5 volumes of ethanol. The eDNA was further purified with a Geneclean® Turbo-Kit (Qbiogene cat #1102-200) following the manufacturer’s protocol, which involves binding eDNA to a resin in a high-salt solution, rinsing with a high-salt buffer followed by elution in a low-salt buffer (1 mM Tris-pH 8.0, 10 mM EDTA). The eDNA quality was assessed by agarose gel electrophoresis and quantity determined by UV spectroscopy on a Nanodrop ND-1000 (Thermo Scientific, DE, USA).

### Library generation

The eDNA from SAE3-0 and SAE3-5 were subjected to Illumina paired-end sequencing protocols at the National Center for Genome Resources (NCGR) in Santa Fe, New Mexico. The purified eDNA was subjected to mechanical fragmentation by nebulization and the resulting double-stranded overhang fragments were end-repaired, phosphorylated, and ligated to proprietary adapter oligonucleotides (Illumina Cat # PE-102-1001). Ligation products were size-selected by gel electrophoresis and the 400 bp fraction excised. Purified DNA libraries were subjected to a final PCR amplification step (10 cycles). The index sequences, oligos six bp in length, were added during the PCR enrichment step in order to allow for the pooling of multiple samples in a single lane of an Illumina IIx flowcell. All amplified libraries are quantitatively and qualitatively assessed by Nanodrop ND-1000 UV/Vis spectroscopy and in a DNA Bioanalyzer 2100 (Agilent, CA, USA).

### Sequencing technology

Ninety bp from each end of the 400 bp fragments were sequenced on the Illumina Genome Analyzer IIx. Sequencing analysis resulted in two files per library, each representing the results from one of each end of the fragment. This sequencing protocol resulted in approximately 7.8 million 90 bp reads of data per file, for a total of 2.8 Gbp of data in a single lane (Table 3). Two files were generated for each sample, one for each end of the molecule.

**Table 3:** Library information

Label	SAE3-0	SAE3-5
Sample Label(s)	SAE3-0	SAE3-5
Sample prep method	G-nome® DNA isolation kit, Geneclean® Turbo-Kit	G-nome® DNA isolation kit, Geneclean® Turbo-Kit
Library prep method	Illumina Paired-End DNA Sample Prep Kit	Illumina Paired-End DNA Sample Prep Kit
Sequencing platform	Illumina IIx	Illumina IIx
Sequencing chemistry	TruSeq SBS v3	TruSeq SBS v3
Sequence size (Gbp)	1.4	1.4
Number of reads	15,503,268	15,877,664
Single-read or paired-end sequencing?	Paired-end	Paired-end
Sequencing library insert size	400	400
Average read length	90	90
Standard deviation for read length	1	1

## Sequence processing, annotation, and data analysis

### Sequence processing

Sequence reads that passed the quality control measures established by Illumina were uploaded to the MG-RAST version 3.0 server [9, 10]. MG-RAST applies additional quality control parameters, determines the guanine and cytosine content (% G + C) for each read, and provides an estimate of sequencing errors using the DRISEE algorithm [11].

### Metagenome processing

MG-RAST compares every read to the non-redundant database M5NR, which includes inferred protein sequences [10]. There are two MG-RAST files for SAE3-0 library that represent each end of the insert; these are EW3Pre1 and EW3Pre2 while the SAE3-5 library files for each end are referred to as EW3Post1 and EW3Post2. The results of sequence processing are shown in Table 4. The metagenome data are available as MG-RAST project number 11259, the North Railroad Avenue Plume EPA Superfund Site.

**Table 4:** Sequence processing

Label	SAE3-0	SAE3-5
Tool(s) used for quality control	MG-RAST (default)	MG-RAST (default)
Number of sequences removed by quality control procedures	1,870,805	1,240,627
Number of sequences that passed quality control procedures	13,632,463	14,637,037
Number of artificial duplicate reads	1,646,292	1,013,551

### Metagenome annotation

The results of MG-RAST phylogenetic analysis include the number reads that match each OTU, and three other attributes: the average percent identity, the average alignment length, and the average expectation (e-value). The cutoff value for the minimum percent identity was 60%, the maximum e-value was 10^−5^, and the minimum average alignment length was 15 Bp. These data were downloaded and read into the statistical software JMP® 11 (SAS, Cary, North Carolina) for distribution, statistical analyses, and plotting.

### Post-processing

The read counts from each end of the DNA fragment identified as a genus-level OTU were used as proxies for replicates providing a measure of the noise in the HTS process. The baySeq algorithm assumes a negative bionomical distribution and uses a Bayesian approach to determine significant differences between high-throughput sequencing data sets and was used to assign FDRs to each genus [12]. Zipf’s law (a.k.a. the power law) [13] was used to determine a cut-off for genera with low frequencies.

Annotations based on metabolic characteristics were based on literature references or the Joint Genome Institute Integrated Microbial Genomes database [14]. Methanogens [15], SRB [16, 17], DHB [18], FeOB [19], SOB [20], and nitrifying bacteria [21] were manually annotated in the dataset.

## Metagenome properties

**Fig. 2:**
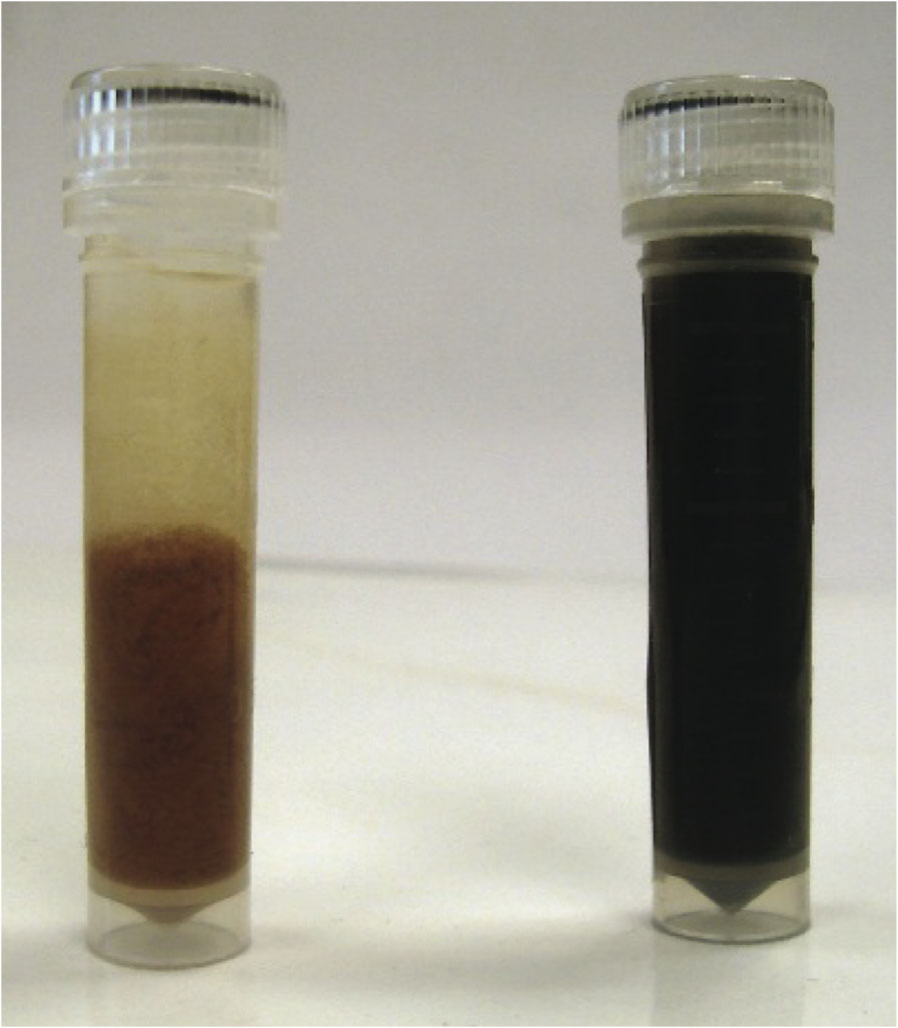
Color shift in microbial samples. The tube on the left is the sample collected one month prior to application of remediation protocols, the tube on the right is the sample collected five months after the addition of EVO, nutrients, and hydrogen gas to the aquifer

The microbial slurries extracted from the filters exhibited a dramatic color shift, from tan in SAE3-0 to black in SAE3-5 (Fig. 2), indicating a shift from an aerobic to anaerobic condition following the addition of the bioamendments. The DNA yield increased from 1.3 to 16.1 μg DNA per L water, indicating a 3.6 fold increase in biomass. A 9% decrease (79 out of 869) in the number of genera annotated was observed after remediation.

The overall properties of the SAE3-0 and SAE3-5 metagenomes are provided in Tables 5, 6, and 7. The level of sequencing errors (the DRISEE score) in the dataset that passed both instrumental and MG-RAST QC metrics falls well within one standard deviation of the 6.4 mean for all the MG-RAST data. Between 86.0% and 97.3% of reads were assigned to an OTU.

**Table 5:** Metagenome statistics

Label	SAE3-0	SAE3-5
Libraries used	SAE3-0	SAE3-5
Assembly tool(s) used	NA	NA
Number of contigs after assembly	NA	NA
Number of singletons after assembly	NA	NA
minimal contig length	NA	NA
Total bases assembled	NA	NA
Contig n50	NA	NA
% of Sequences assembled	NA	NA
Measure for % assembled	NA	NA

**Table 6:** Annotation parameters

Label	SAE3-0	SAE3-5
Annotation system	MG-RAST	MG-RAST
Gene calling program	FraGeneScan	FraGeneScan
Annotation algorithm	MG-RAST	MG-RAST
Database(s) used	MNR5	MNR5

**Table 7:** Metagenome properties

Label	SAE3-0	SAE3-5
Number of contigs	NA	NA
Gbp	1,226,921,670	1,317,333,330
Number of features identified	6,589,534	6,873,193
CDS	4,786,768	5,056,492
rRNA	1,802,766	1,816,701
others	0	0
CDSs with COG	1,468,398	1,648,018
CDSs with Pfam		
CDS with SEED subsystem		
Alpha diversity	217.523	196.901

**Fig. 3:**
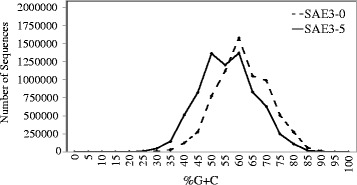
Percent G + C shift at NRAP. A major peak at 60% is observed prior to remediation, but a bimodal distribution is observed with peaks at 45% and 60% G + C after bioamendment application

A distinctive shift toward decreased %G + C content is evident following biostimulation (Fig. 3). One explanation is that this change indicates shift toward species with lower %G + C, but it may also reflect recombinational processes during microbial evolution that facilitates horizontal transmission of genes. For example, *Dehalococcoides* codons tend toward high %G + C content, but the genes specific for VC respiration are composed of codons with low %G + C content that are flanked by mobile genetic elements, indicating that these genes are a relatively recent acquisition of the *Dehalococcoides* genome [22]. The biological significance of the shift in %G + C content at NRAP remains to be determined, but similar changes are observed in other microbial communities [23].

### Taxonomic diversity

The phyla level taxonomic diversity at NRAP is shown in Table 8. All taxa detected by DGGE (*Dechloromonas* *,* *Sulfurimonas* *,* *Thiomicrospira* *,* *Sulfurovum* *,* *Gallionella* *, and* *Zoogloea*) and PLFA (*Firmicutes* *,* *Proteobacteria* *,* Anaerobic Metal, SRB/*Actinomycetes* *)* are detected in both metagenomes. Data obtained from Microbial Insights using qPCR indicate increases in *Dehalococcoides* *,* *Desulfuromonas*, and *Dehalobacter*, but *Dehalobacter* is missing from the metagenomic dataset that relies on protein features for annotation, although a few matches (<10) to *Dehalobacter* are detected as RNA features in SAE3-5. *Dehalobacter* and *Dehalococcoides* are known to compete with each other [24], making it possible that population fluctuations rendered *Dehalobacter* barely detectable when the metagenomic samples were collected. In addition, *Dehalobacter* may not be required for successful remediation, as it was not detected in DehaloR^2; a TCE dechlorinating consortium isolated from sediment and analyzed by 16S rRNA and qPCR [25]. *Dehalobacter* abundance and its role in this successful remediation project are under investigation.

**Table 8:** Taxonomic composition

Phylum	SAE3-0 CPM^a^	SAE3-5 CPM	Log_2_ Fold-Change	Total Raw Counts
*Proteobacteria*	902,173.65	777,848.47	−0.21	14,270,944
*Firmicutes*	18,007.05	56,618.88	1.65	665,155
*Euryarchaeota*	2,431.96	64,504.59	4.73	616,044
*Bacteroidetes*	14,088.27	33,537.14	1.25	420,818
*Actinobacteria*	17,115.85	10,802.99	−0.66	234,150
*Cyanobacteria*	11,765.89	11,318.40	−0.06	196,982
*Chlorobi*	4,290.76	6,684.93	0.64	95,503
*Planctomycetes*	6,289.51	2,718.90	−1.21	74,466
*Chloroflexi*	4,178.12	4,307.47	0.04	72,617
*Verrucomicrobia*	3,266.26	2,962.45	−0.14	53,021
*Acidobacteria*	2,671.54	2,687.13	0.01	45,811
unclassified (derived from *Bacteria*)	2,923.61	1,836.12	−0.67	39,911
*Spirochaetes*	1,307.70	3,105.62	1.25	38,993
*Aquificae*	952.30	2,758.34	1.53	32,993
*Deinococcus-Thermus*	1,709.80	1,658.48	−0.04	28,752
*Thermotogae*	8,47.05	2,259.90	1.42	27,555
*Nitrospirae*	1,631.34	1,515.31	−0.11	26,812
*Deferribacteres*	497.52	2,447.58	2.30	26,552
*Synergistetes*	430.03	2,233.53	2.38	24,042
*Fusobacteria*	482.21	2,029.32	2.07	22,561
*Chrysiogenetes*	301.95	1,136.92	1.91	12,889
*Lentisphaerae*	257.31	1,129.68	2.13	12,472
*Chlamydiae*	514.61	618.05	0.26	9,754
*Crenarchaeota*	371.35	654.25	0.82	8,966
*Elusimicrobia*	146.83	828.32	2.50	8,817
*Gemmatimonadetes*	471.62	239.12	−0.98	5,910
*Tenericutes*	198.75	394.06	0.99	5,205
*Dictyoglomi*	132.67	356.46	1.43	4,339
unclassified (derived from *Archaea*)	72.84	344.47	2.24	3,759
*Fibrobacteres*	81.77	232.31	1.51	2,791
*Thaumarchaeota*	226.18	104.16	−1.12	2,737
Candidatus *Poribacteria*	135.61	71.53	−0.92	1,725
*Korarchaeota*	22.07	46.03	1.06	599
*Nanoarchaeota*	6.00	9.08	0.60	131

^a^CPM: Counts per million. Normalized values were calculated by dividing the abundance for each genera by the column total and multiplying by 10^6^.

Members of the genus *Geobacter* exhibit a dramatic increase in abundance, increasing from 0.8 to 21% of all prokaryotes. This group was not measured as part of qPCR, DGGE, or PLFA experiments. *G. lovleyi* is an iron-reducing dechlorinator [26] that becomes the dominate species in response to ERD (Fig. 5). *G. lovleyi* and other members of this genus, including *G. sulfurreducens* and *G. uraniireducens*, are capable of transmitting electrons over special pilli known as microbial nanowires [27]. These species are capable of direct interspecies energy transfer, a recently recognized characteristic of biofilms capable of bioremediation [28, 29]. *Geobacter* as well as SRB are detected during uranium bioremediation [30]. *G. lovleyi* can transfer cobamide to *Dehalococcoides* in culture, indicating that these species form an important metabolic link during ERD [31].

*Dehalococcoides mccartyi* are the only microbes currently known to fully respire PCE to ethene [32]; this species exhibit a 0.5 log_2_ fold change increase in abundance at NRAP, an outcome consistent with effective ERD [25]. Matches to *D. mccartyi* strains 195, GT and VS, CBDB1, and BAV are detected at NRAP. This finding is consistent with pangenomic microarray results for *Dehalococcoides* enrichment cultures, in which heterogeneous mixtures of reductive dehalogenase genes are found, indicating the presence of a mixture of species with dehalorespiration abilities [33, 34]. A dechlorinating enrichment cultures is known to contain a variety other phylogenetic groups [35], all of which increase at NRAP. Following six months of ERD at the NRAP site vinyl chloride was just beginning to increase (Fig. 1), so the small increase in *Dehalococcoides* is consistent with contaminant levels and it is expected that *Dehalococcoides* will increase in subsequent sampling events.

*Dehalogenimonas lykanthroporepellens* is another DHB of the phylum *Chloroflexi* that exhibits a similar level of increase as other members of *Dehalococcoidia* at NRAP. *D. lykanthropore* was first isolated from a chlorinated solvent Superfund site in Louisiana [36, 37] and has been detected in contaminated aquifers in Europe [38]. Other dehalorespiring species include the *Firmicutes* *Desulfitobacterium* and the *Deltaproteobacteria* *Desulfovibrio* *;* both increase in abundance at NRAP, consistent with findings from RD enrichment cultures [39].

The increase in *Epsilonproteobacteria* is lead by the genus *Sulfurospirillum*, which exhibits the largest increase (8.2 log_2_ fold change) in abundance in response to ERD. Of the two species OTUs found at NRAP, one is capable of halorespiration (“*S. multivorans* *”*) and is found at other chlorinated solvent contaminated sites [40]. Other chemolithotropic *Epsilonproteobacteria* genera that increase in response to the bioamendment are *Sulfuricurvum*, a sulfur-oxidizing facultative anaerobe first isolated from oil-storage containers [41] as well as *Caminibacter* [42], *Nitratifractor* [43], *Nautilia* [44], and *Sulfurovum* [45]; each of which were first isolated from deep-sea hydrothermal vents. The pathogenic branch of *Epsilonproteobacteria* is also represented at NRAP and provides additional evidence for groundwater as a reservoir for emerging pathogens [45]. Moreover, and of concern in the design, operation, and monitoring of an ERD remediation process, these potential pathogens were seen to increase in abundance following the addition of bioamendments (Fig. 5d). Two similar species that were observed to increase in abundance are *Arcobacter nitrofigilis* [46], which is a non-pathogenic nitrogen-fixing species and *Arcobacter butzleri* *,* known to cause diarrhea in humans [47]. Other examples of pathogenic OTUs that increased in abundance following the addition of bioamendments include *Campylobacter jejuni* *,* a well-characterized food and water-borne pathogen [48] and *Helicobacter pylori*, which is linked to gastric ulcers and other gastrointestinal syndromes [49]. Although *Epsilonproteobacteria* play an important role in the bioremediation process, these data indicate that testing for pathogenic bacteria is warranted when certifying that a formerly contaminated water source that has undergone treatment by ERD is safe.

The genus *Desulfuromonas* includes dehalorespiring species, although none of the species detected at NRAP are known to have this ability. *De novo* alignment and annotation is necessary to determine if a new dehalorespiring strain can be identified.

“*Anaeromyxobacter dehalogenans*” is one of three species of this *Deltaproteobacteria* genus detected at NRAP and it is capable of aryl-halorespiration. Matches to the L-haloacid dehalogenases present in the genomes of *Pyrococcus horikoshii* [50] and *Sulfolobus tokodaii* [51] are detected at NRAP. As members of thermophilic *Archaea*, these are unlikely matches since the average groundwater temperature at NRAP during the sampling period was 18.6 °C, far below the range for thermophiles. Additional data collection is underway to facilitate *de novo* alignment of NRAP genomes and to identify alternative dehalogenation pathways.

In addition to the *Deltaproteobacteria* and phyla *Chloroflexi* and *Firmicutes*, methanogenic *Archaea* carry out key steps in the metabolic pathways during RD [33] and all three phylogenetic groups increase during the first five months of remediation. The dramatic increase in methanogenic *Archaea* (Fig. 5c) also explains the increase of on-site methane concentrations in the shallow vadose zone, an important consideration for safe ERD design and operation.

### Functional diversity

Table 9 lists the functional diversity of the metagenomes from a protein-centric perspective using COG annotations. The availability of qPCR, PLFA, and DGGE data collected to determine the optimal remediation protocol provides evidence for the functional diversity of microbes, not just the genes. This facilitates a focus on shifts in taxonomy that occur in response to bioremediation that led to the degradation of PCE.

**Table 9:** Functional diversity

COG Category	Metagenome SAE3-0	Metagenome SAE3-5
Cellular processes and signaling	337,528	395,668
Information storage and processing	303,004	291,760
Metabolism	650,216	750,444
Poorly characterized	177,649	210,146

### Statistical and visualization results

**Fig. 4:**
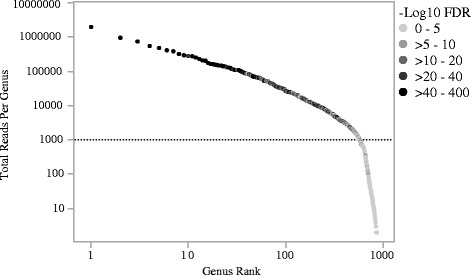
Zipf curve. Plotting the log reads per genus against the genus rank reveals an inflection point that separates genera with coverage within a linear from those with limited coverage. Genera with less than 1000 reads were excluded from subsequent analyses

It can be difficult to determine an appropriate level of coverage for further examination. Here we turn to the Zipf’s law and plot the log_10_ reads per genus against the log_2_ of the genus rank and annotate the with the -log_10_ FDRs (Fig. 4). A clear inflection marks delineates where a logical cut-off can be made. For our preliminary analyses, genera with a total of 1000 counts or greater chosen for further annotation and visualization. This filtering step results in 579 genus-level OTUs retained of the 869 total (67%).

**Fig. 5:**
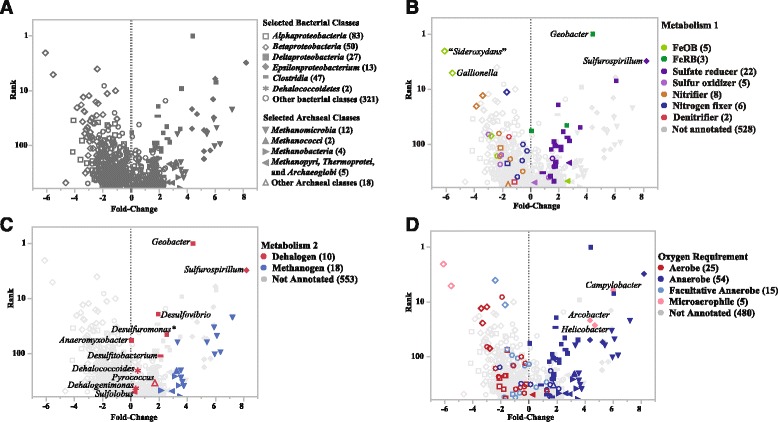
Rank abundance fold-change plot of genera detected at the NRAP site. Each of the 569 genera has at least 1000 reads and are ranked by their abundance on the y-axis. On the x-axis, those to the left of zero decrease in abundance after six months, those to the right increase. The number of genera in each category is indicated in parentheses. The labeled genera are discussed in the text. **a** Selected classes are indicated by marker shape. **b** The metabolic classification of genera is indicated by color. **c** Methanogenic and dehalogenic bacteria are highlighted by color. **d** Oxygen requirements for selected genera are indicated by color and demonstrates that the remediation protocol selects for anaerobic species. The labeled species are potential pathogens

To visualize the ecological shifts in the community, we employ a modified rank abundance curve, in which the abundance variable is replaced by log_2_ fold-change (Fig. 5) for each prokaryotic genera. Using this convention, the genera that increase as a result of biostimulation are located to the right of 0, those that decrease graph to the left. All genera are detectable before remediation, indicating that any non-indigenous bacteria introduced into the aquifer by injection of bioamendments have no affect on the ecology. Seventy-nine genera are not detected after remediation protocols, this decrease in diversity is expected as the community adapts to the availability of EVO as carbon source. The shape of the markers indicates the taxonomic affiliation of selected genera (Fig. 5a) highlights the phylogenetic shift in the community in response to remediation. Although the proteobacteria exhibit little change overall (Table 8), 81 out of 83 of *Alphaproteobacteria*, all 50 *Betaproteobacteria*, and all 89 *Gammaproteobacteria* genera decrease in abundance. Twenty-one out of 27 *Deltaproteobacteria* and all 13 *Epsilonproteobacteria* are more abundant after remediation. The expansion of *Archaea* is dominated by increases in methanomicrobia (12/12).

The phenotypic characteristics of interest in bioremediation are polyphyletic, so it is necessary to consider relevant metabolic classifications independent of taxon. The rank fold-change plot can be further annotated to visualize multiple metabolic attributes of the data. In Figs. 5b-5d, selected metabolic categories are highlighted by color and facilitate the correlation of changes in groundwater chemistry to the taxonomic changes in the microbial community. The increase in ferrous iron detected in source area groundwater following the addition of EVO (Table 10) is consistent with the decrease in FeOB and increase in the FeRB (Fig. 5b). The surge in SRB reflects the decrease in sulfate and explains the unpleasant odor encountered during the November sampling. Genera that include species capable of dehalogenation and methanogenesis are notated by color in Fig. 5c; both increase. The shift toward anaerobic conditions in the aquifer is confirmed in Fig. 5d, in which the oxygen requirement of genera for which data are available are indicated by color.

**Table 10:** Geochemical Parameters and Contamination Concentrations

Classification	Parameter	Units	SAE3-0 ^a^ Mean ± SD	SAE3-5 ^b^ Mean ± SD	Log_2_ Fold-Change
Dissolved Gas by Headspace	Methane	μg/L	BDL^c^ (<1.0)	6540.0 ± 4292.8	13.7
	Ethene	μg/L	BDL (<2.0)	BDL (<2.0)	0
	Ethane	μg/L	BDL (<2.0)	BDL (<2.0)	0
Anions	Chloride	mg/L	73.7 ± 1.4	83.4 ± 4.7	0.2
	Bromide	mg/L	2.66 ± 2.21	2.48 ± 0.37	−0.1
	Nitrogen, Nitrate (as N)	mg/L	2.05 ± 0.15	BDL (<0.26)	−4.0
	Sulfate	mg/L	213.3 ± 5.2	27.5 ± 30.5	−3.0
Metals	Dissolved Fe	mg/L	0.22 ± 0.08	9.66 ± 9.93	5.5
	Total Mn	mg/L	0.19 ± 0.04	7.54 ± 0.48	5.3
Volatiles	PCE	μg/L	9966.7 ± 1672.9	157.2 ± 196.3	−5.9
	TCE	μg/L	74.7 ± 38.5	223.4 ± 249.6	1.6
	Cis-1,2-DCE	μg/L	390.0 ± 114.7	7840.0 ± 3266.2	4.3
	Trans-1,2-DCE	μg/L	BDL (<125)	143.40 ± 62.90	6.8
	Vinyl chloride	μg/L	BDL (<50)	53.6 ± 21.5	6.7
	Total VOCs	μM	61.248 ± 10.298	85.892 ± 32.489	0.5
TOC	TOC	mg/L	2.13 ± 0.08	104.40 ± 28.95	5.6
Alkalinity	Total (as CaCO_3_)	mg/L	311.7 ± 4.1	684.0 ± 89.9	1.1
	Carbonate	mg/L	BDL (<2.0)	BDL (<2.0)	0
	Bicarbonate	mg/L	311.7 ± 4.1	684.0 ± 89.9	1.1
	Hydroxide	mg/L	BDL (<2.0)	BDL (<2.0)	0
	Sulfide	mg/L	BDL (<1.0)	3.28 ± 1.79	2.7
CO_2_	Total CO_2_	mg/L	291.7 ± 7.5	716.0 ± 103.6	1.3
Field Data	Temperature (°C)	°C	16.723 ± 1.276	20.118 ± 1.844	0.3
	Dissolved O_2_	mg/L	0.227 ± 0.166	0.074 ± 0.057	−1.6
	pH		7.250 ± 0.301	7.270 ± 0.334	0.004
	Oxidation-reduction potential		−12.97 ± 46.96	−247.72 ± 44.07	−4.3
	Conductivity	ms/cm3	1.1923 ± 0.1222	1.6318 ± 0.1367	0.5

^a^Each value is the average of six geochemical sampling events prior to sampling.

^b^Each value is the average of five geochemical sampling events in Nov. 2007, prior to sampling.

^c^One-half of the BDL divided by the dilution factor was used to calculate fold-change. For trans-1,2,-DCE and VC the sample dilution factor was 50. No dilution was used for all other BDL sample results (dilution factor = 1)

## Conclusions

We demonstrate the efficacy of whole metagenome sequencing as a tool to understand ERD and developed a statistical approach and a visualization method that aids this goal. The major difficultly with annotating plots with metabolic data is the lack of a single, authoritative source for metabolic characteristics of bacterial species. Most of the annotation was done manually by consulting the literature. Although the Joint Genome Institute provided a method of annotating genomes with metabolic classifications, few of the genomes detected are annotated by metabolic data. Despite this issue, it is clear that geochemical changes are correlated to the shift in the microbial community. Taxa capable of dehalorespiration and methanogenesis increase, as do species that thrive in anaerobic conditions, supporting the established principles of ERD. The importance of *Geobacter* in bioremediation is confirmed by NRAP data. Additionally, there are findings from these metagenomes that suggest topics for future discoveries. The NRAP data suggests that uncharacterized *Archaea* may also play a role in successful bioremediation of chlorinated solvents. The presence of pathogenic OTUs indicates that testing prior to re-certification of aquifer as potable is a concern. As additional data is obtained from NRAP, *de novo* alignment and annotation of NRAP genomes will increase our understanding of ERD and its role in biodegradation.

## Abbreviations

BDL: Below detectable limits
DCE: Dichloroethene
DGGE: Denatured gradient gel electrophoresis
DHB: Dehalogenating bacteria
DRISEE: Duplicate read inferred sequencing error estimation
eDNA: Environmental DNA
EPA: Environmental Protection Agency (Washington DC, USA)
ERD: Enhanced reductive dechlorination
EVO: Emulsified vegetable oil
FDR: False discovery rate
FeOB: Iron oxidizing bacteria
FeRB: Iron reducing bacteria
Gbp: Giga base pairs
ITRC: Interstate Technology and Regulatory Council (Washington, DC, USA)
MG-RAST: Metagenome rapid annotation using subsystem technology
NA: Not available
NRAP: North Railroad Avenue Plume
OTU: Operational taxonomic unit
PCE: Tetrachloroethene (a.k.a. perchloroethylene)
PLFA: Phospholipid fatty acids
qPCR: Quantitative polymerase chain reaction
RD: Reductive dechlorination
SAE3-0: Source Area extraction well 3, zero months
SAE3-5: Source area extraction well 3, five months
SOB: Sulfur oxidizing bacteria
SRB: Sulfate reducing bacteria
TOC: Total organic carbon
VC: Vinyl chloride
VOCs: Volatile organic carbon
